# Supplementary material for: Are carbon emissions trading and green financial instruments synergistic? -Comprehensive quantitative research based on content analysis
Source: PLoS One. 2024 Mar 7;19(3):e0298601. doi: 10.1371/journal.pone.0298601 (PMC10919601; doi:10.1371/journal.pone.0298601)
Supplement: S1 Data — (ZIP) [file pone.0298601.s001.zip › Data/地级市Z_F工作报告.zip!/╜¡╦╒/─╧╛⌐╩╨/└┤╫╘╣1⁄2╓┌║┼í╛┬φ┐╦╩2╛▌═°í┐_2018.docx]

各位代表：

　　现在，我代表南京市人民政府，向大会报告工作，请予审议，并请政协委员和其他列席人员提出意见。

　　一、本届政府工作回顾

　　市十五届人大一次会议以来，在党中央国务院、省委省政府和市委坚强领导下，我们认真贯彻党的十八大、十八届三中、四中、五中、六中全会和十九大精神，以习近平新时代中国特色社会主义思想为指导，主动适应经济发展新常态，坚持稳中求进工作总基调，以供给侧结构性改革为主线，统筹推进稳增长、促改革、调结构、惠民生、防风险各项工作，加快建设“强富美高”新南京，圆满完成“十二五”规划和本届政府任期各项目标任务，实现“十三五”良好开局，各项工作开创了新局面。

　　（一）经济建设取得重大成就。五年来，我们始终坚持发展第一要务，在优化结构中做大总量，在提升效益中加快发展，规模总量和质量效益实现“双提升”。经济保持中高速增长。全市地区生产总值连跨4个千亿台阶、达到11715亿元，成为全国第11个突破万亿规模的城市，年均增长9.3%。人均地区生产总值突破2万美元，保持全国前列。一般公共预算收入连跨5个百亿台阶，总量达到1272亿元，年均增长11.7%。全市金融机构本外币存款余额、贷款余额分别突破3万亿元和2.5万亿元，均居全省第一。固定资产投资突破6000亿元，社会消费品零售总额、民营经济增加值均突破5000亿元。产业转型升级取得重大进展。服务业主导地位更加凸显，河西CBD等一批现代服务业集聚区建设提速，区域金融中心、长江航运物流中心建设取得阶段性成效，服务业增加值占地区生产总值比重达到59.7%，现代金融、文化创意等高端服务业成为支柱产业。战略性新兴产业加快发展，主营业务收入年均增长13%，占规模以上工业比重达到40%，千亿级新兴产业集群达到6个，高世代液晶面板等一批重大项目竣工投产，台积电等一批龙头项目加快建设，集成电路及专用设备、新型显示、智能电网、新能源汽车等新兴产业链加速形成。传统产业加快改造提升，化工、钢铁等高耗能产业占规模以上工业总产值比重从34.4%下降到31.9%。现代农业建设水平稳步提升，成功创建国家现代农业示范区，农业基本现代化建设水平跃居全省第一，都市现代农业发展水平居全国第三。创新驱动发展战略深入实施。高新技术企业达到1850家，全社会研发经费支出占地区生产总值比重、每万人发明专利拥有量均保持全省第一、跻身全国前列。苏南国家自主创新示范区联动发展格局初步形成，新增高淳、麒麟、白马、徐庄四家省级高新区，全市各区实现高新园区全覆盖。“两落地一融合”工程稳步推进，省产业技术研究院、中德智能制造研究院等一批新型研发机构签约落地。国家未来网络试验设施、第五代移动通信等重大科技创新平台建设加快推进。“创业南京”英才计划深入实施，集聚科技顶尖专家33名、培育创新型企业家117名、引进高层次创业人才1280名，引领10894名青年大学生创业，21名企业人才入选国家“万人计划”，在省内和副省级城市中位居第一。建立完善科技奖励评价办法，科技创新券、风险补偿、创投引导等政策措施取得明显成效。设立中国（南京）知识产权保护中心，成为全国首批知识产权强市创建市。“三去一降一补”工作扎实推进。轧钢、水泥、船舶产能累计减少200万吨、572万吨、44万载重吨，房地产市场调控取得阶段性成效，累计置换政府债券1696亿元，降低实体经济企业成本超过680亿元，120个补短板项目完成投资1490亿元。成功创建“全国质量强市示范城市”。

　　（二）城市功能品质显著提升。五年来，我们认真贯彻中央城市工作会议精神，扎实推进城市规划建设管理各项工作，城市布局更加优化、功能更加完善、面貌焕然一新。南京被国家定位为东部地区重要中心城市、长三角特大城市。空间格局更加优化。江北新区获批为国家级新区并全面启动规划建设，河西新城呈现现代化城市中心形象，南部新城完成机场搬迁并实质性启动，麒麟高新区、江心洲生态科技岛建设取得积极进展，东山、仙林、高淳、溧水副城功能加快完善，“多心开敞、轴向组团、拥江发展”的现代都市区空间格局基本形成。新型城镇化加快推进，城市建成区面积由619平方公里拓展到773平方公里，城镇化率达到82.3%。枢纽功能更加凸显。宁杭高铁、宁安城际相继开通，宁启铁路复线电气化改造完成，溧马、淳芜高速公路建成投用。长江12.5米深水航道初通南京，禄口机场二期、小红山客运站等一批重要枢纽工程建成投用。路网体系加快完善。机场高速扩建、江北大道、宁高新通道、122省道等一批城乡大通道竣工投用。城西干道和江东路快速化改造、纬七路东进等一批城市骨干道路工程建成通车，“井字加外环”快速路网基本成型。过江通道建设全面提速，建成扬子江隧道，开工建设长江五桥、和燕路过江通道，长江大桥公路桥维修改造有序推进，长江隧道、扬子江隧道免费通行。提档升级农村公路2108公里。公共交通全国领先。轨道交通实现网络化运行，7线263公里轨道交通线路新投入运营，运营总里程达到348公里、居全国第四。建成河西、麒麟两条有轨电车线路，更新新能源公交车4100余辆，新增公共自行车9.4万辆，公交线网城乡全覆盖，主城区公交机动化出行分担率达63.1%，创成首批“国家公交都市示范城市”。历史文化保护取得明显成效。制定实施近现代建筑保护和利用三年行动计划，推进明城墙保护、整治、开放与联合申遗工作，南京被国家确定为“海上丝绸之路”申遗城市，建成牛首山文化旅游区、大报恩寺遗址公园、六朝博物馆等一批重点文化项目，梅园新村、颐和路入选全国首批30条历史文化街区，沿明城墙、秦淮河、长江、历史街巷生态人文空间特色彰显。市容环境展现新面貌。大力实施城市精细化建设管理专项行动，完成56条主次干道、869条背街小巷环境综合整治，禄口机场、南京南站、玄武湖周边等窗口地区提档升级。完成老旧小区整治587个，受益居民19.7万户。改革停车管理政策，累计新增停车泊位超过9万个。落实“门前三包”责任制，城区道路机扫率达90%，提高30个百分点。高标准通过国家卫生城市复审，再次荣获“全国文明城市”荣誉称号。防洪保安能力大幅提升。长江干堤、滁河、水阳江等主要江河干流防洪能力显著增强，防洪减灾安全保障体系更加完善。成功应对2015、2016年特大汛情。

　　（三）生态文明建设成效明显。五年来，我们切实践行绿色发展理念，大力推进国家生态文明先行示范区建设，扎实开展“两减六治三提升”专项行动，生态环境质量明显好转，成功创建“国家森林城市”“国家生态市”。打出大气污染防治“组合拳”。深入实施工业废气治理，完成电力燃煤机组超低排放改造，全面开展挥发性有机物治理；实施车辆、油品“双提标”，完成黄标车淘汰任务；狠抓工地扬尘管理，全面推行烟花爆竹“禁放”，严格落实冬春季节大气管控措施。2017年，全市PM2.5平均浓度比2013年下降46.8%，空气质量优良天数比例提升到72%以上，PM2.5浓度降幅、空气质量优良天数改善幅度均居全省首位。实施水环境治理“攻坚战”。全面推进“河长制”和“断面长制”，完成150条黑臭河道整治，基本消除建成区黑臭水体，主城沿河环境明显提升；建成铁北、珠江二期、城东三期等一批污水处理厂及10个镇（街）、1296个规划布点村污水处理设施，完成277平方公里雨污分流，城镇污水处理率达94.5%；长江、太湖流域保护深入推进，主要集中式饮用水水源地水质达标率保持100%。推进节能减排降耗“硬举措”。积极推进四大片区工业布局调整，关停整治“三高两低”企业609家，煤炭消耗总量得到有效控制，全市万元GDP能耗较2012年下降24.3%。环卫基础设施加快建设，建成江南、江北、高淳等一批垃圾焚烧发电设施，生活垃圾无害化处理率达到100%。加强生态文明制度建设，实施排污许可证管理、排污权交易和排污收费制度改革，通过经济手段引导企业治污减排。开展国家低碳城市试点工作。打造生态建设“风景线”。划定生态红线保护区域。出台实施南京“土十条”，土壤环境修复工程进展顺利。在全省率先完成农村环境连片整治，建成美丽乡村示范区2000多平方公里、市级以上示范村486个。建成滨江风光带、青奥森林公园等一批重点生态工程，新增绿地小游园99个，新建绿道562.6公里。绿化造林10万亩，城市绿化覆盖率、林木覆盖率分别达到44.8%和29.9%。

　　（四）改革开放取得重大突破。五年来，我们全面深化改革，不断提升开放水平，发展动力活力显著增强。行政审批制度改革进一步深化。加大“放管服”改革推进力度，建设“互联网+政务服务”体系，公布行政权力十张清单，取消行政审批事项129项，7次向基层下放审批事项326项，703项非行政许可审批全部取消，成为全国第二个没有非行政许可审批城市。各类市场主体活力不断增强。新增41家上市企业、237家挂牌企业，上市企业总数达到102家。苏宁银行成为省内首家民营银行。新组建扬子、东南、体育、旅游等国有企业集团，62家企业完成混合所有制改革，市属经营性资产证券化率达到52.2%。综合医改稳步推进。实施医疗医保医药“三医”联动，全市各级各类公立医疗机构全面实施药品零差率销售，医疗保险报销比例和大病保险支付水平大幅提高，医疗联合体、家庭医生、智慧医疗成为南京医改特色品牌。改革试点工作成效显著。贸易流通体制改革等21项国家和省级改革试点取得阶段性成效，一批改革亮点和品牌加快形成，成立全国第一家具备综合功能的文化金融服务中心，“不见面审批”模式在全省推广并形成广泛影响。“一窗受理、集成服务”不动产统一登记全面推行。在全省率先完成农村土地承包经营权确权登记颁证。全面完成村组集体资产清产核资，为全国提供了“南京方案”。商事制度、户籍制度、综合执法、价格机制、生态文明体制等重要领域和关键环节改革均取得积极进展。大力推动对外开放。积极参与“一带一路”国际合作，南京综合保税区、跨境电子商务产业园等一批开放平台获批运行，16条国际航线、南京—中欧等洲际集装箱货运班列开通运行，江心洲生态科技岛、空港经济开发区获批为省级开发区。实际利用外资在全省份额由11.6%提升至14.4%，对外贸易结构更加优化，一般贸易进出口占全市进出口比重提高8个百分点，服务外包综合排名全国第一，国际经济技术合作迈上新台阶。区域合作加快推进。积极参与长江经济带建设，充分发挥在扬子江城市群建设中的龙头带动作用，宁镇扬一体化和南京都市圈建设取得实质性进展，宁杭生态经济带建设协同推进。城市国际影响力显著提升。南京青奥会精彩圆满，赢得了海内外高度评价；国家公祭活动顺利顺畅，展现了南京开放自信、和平博爱的国际形象。成功举办亚青会、速度轮滑世锦赛、全项目轮滑世锦赛、南京马拉松等体育赛事，全球体育城市指数排名从第42位跃升到第19位。举办两岸企业家紫金山峰会、世界智能制造大会等一批重大活动，提升了南京的知名度和影响力。

　　（五）人民生活不断改善。五年来，我们深入贯彻以人民为中心的发展思想，围绕“八个更”要求，狠抓民生保障改善，全市财政一般公共预算支出中民生支出占比达78%，连续9年入选中国“最具幸福感城市”。富民增收步伐加快。多措并举充实群众“口袋”，城乡居民人均可支配收入分别达到54538元和23133元，年均分别增长8.9%和10.1%。精准扶贫、精准脱贫顺利推进，全市80%低收入农户和60%以上经济薄弱村实现脱贫摘帽。实施积极的就业创业政策，推动实现更加充分、更高质量就业，累计培育自主创业者8.74万人，创业带动就业62.85万人，城镇新增就业110万人，城镇登记失业率控制在2.5%以内，低于全省平均水平1个百分点。基本公共服务均等化水平大幅提升。教育资源配置趋于均衡，全面普及15年基础教育，小学全面实施“弹性离校”，在全省率先实行学前一年免费教育，新增幼儿园学位8万多个，义务教育优质资源覆盖率达到92%，全市域创成“全国义务教育均衡发展区”，教育现代化水平位居全省第一。医疗卫生事业加快发展，河西儿童医院、市公共卫生医疗中心投入运行，市中医院新院区试运行开诊，新城新区公共卫生资源布局不断完善。居家养老服务、社会化养老全面推行。大力发展文化事业，话剧《雨花台》等7部作品获全国精神文明建设“五个一工程”大奖，成功举办第八届中国京剧艺术节等活动，基本建成四级公共文化服务体系，建成首批“江苏省书香城市建设示范市”。大力开展全民健身，全市域创建成为省公共体育服务体系示范区。智慧城市建设步伐加快，市民卡、“我的南京”APP、智慧医疗等服务广泛应用，全市公共区域WiFi网络基本实现免费开放。社会保障网络更为健全。在全省率先实现“五险”市级统筹，被征地人员社会保障制度、城乡统一的居民基本养老保险制度全面建立，社保覆盖面持续扩大，待遇稳步提高。城镇职工五项险种累计参保五年净增209万人次，城乡基本养老保险、医疗保险和失业保险覆盖率均保持在98%以上，企业退休人员月人均养老金水平提高到2908元、居全省第一。住房保障力度进一步加大，累计完成棚户区改造1696万平方米，竣工各类保障性住房2058万平方米，受益居民18.8万户，既有住宅增设电梯取得突破性进展，为3.87万名高校毕业生发放租房补贴。城乡居民“米袋子”“菜篮子”得到有效保障，完成176个农贸市场提档升级。社会治理体系更加完善。创新社区治理和服务，荣获“全国和谐社区建设示范市”称号。社区“减负增效”、街道社区体制改革等，被评为全国社区治理十大创新成果。平安南京、法治南京建设水平不断提升，各项重大安保任务圆满完成。健全完善拥军系列政策，青年大学生应征入伍率连续三年全国领先，荣获全国双拥模范城“八连冠”。安全生产、食品药品安全、信访稳定等各项工作扎实推进，民族、宗教、档案、史志、外事、侨务、社会科学工作取得新进展，妇女、儿童、仲裁、残疾人、慈善事业取得新业绩。民生实事项目全部兑现。我们始终把为民办实事作为政府的一项重点工作，实行清单化管理、项目化推进，每一年度民生实事项目全部完成，群众得到了更多实惠。

　　五年来，我们扎实抓好职能转变、作风改进、制度建设和反腐倡廉等各项工作，政府公信力和执行力不断增强。对全市重点目标任务实行“挂图作战”，打造“网上批、快递送、不见面”政务服务新模式，推行投资项目“预审代办制”，行政效能持续提升。坚持把纪律和规矩挺在前面，严格执行中央八项规定和省委市委作风建设相关要求，防止“四风”问题反弹回潮。认真贯彻国务院“约法三章”要求，全市“三公”经费支出降低55%左右。制定实施《关于深入推进依法行政加快建设法治政府的意见》，荣获全国首批“法治政府建设典范城市”称号。主动接受人大法律监督和政协民主监督，累计办理市人大代表建议1763件、政协提案3131件，提请市人大常委会审议地方性法规草案27件，制定修改政府规章54件。落实全面从严治党要求，扎实开展党的群众路线教育实践活动和“三严三实”专题教育，推动“两学一做”学习教育常态化制度化。坚决落实党风廉政建设“两个责任”，保持了良好的政治生态和干事创业氛围。

　　各位代表，刚刚过去的五年，是全方位变革的五年，是开创性奋进的五年。五年的变化有目共睹，五年的奋斗凝聚艰辛，五年的成绩来之不易。这些成就，归功于党中央国务院和省委省政府以及市委的坚强领导，归功于市人大、市政协和社会各界的支持监督，归功于全市人民的团结奋斗、拼搏奉献。在这里，我代表南京市人民政府，向在各个领域和岗位上辛勤劳动的全市人民，向给予我们支持与监督的人大代表、政协委员，向各民主党派、工商联、人民团体以及社会各界人士，致以崇高的敬意！向大力支持我们工作的人民解放军和武警驻宁部队、中央和省驻宁机构和单位，向关心和支持南京改革发展的港澳台同胞、海外侨胞、国际友人，表示衷心的感谢！

　　在总结成绩的同时，我们也清醒地认识到，全市经济社会发展和政府工作中还存在一些困难和问题。主要是：发展质量和效益还不高，产业转型升级任务艰巨，规模以上工业企业数量较少，在全国、全球影响力强的领军企业不多；实体经济发展仍然面临一些突出困难，民营经济发展还不充分，小微企业成长环境仍需改善；科技体制改革力度需要进一步加大，科教资源优势还没有充分发挥，企业创新主体地位尚不突出，创业投资等新金融要素不够活跃，科技成果落地转化还有很大空间；城乡区域发展不平衡现象依然存在，城乡居民收入落差还比较大；城乡环境综合整治还需进一步加强，空气环境、水环境质量需要持续提升，资源能源总体消耗水平过高，城市管理的科学化、精细化水平有待提高；公共服务和社会保障水平需要提升，优质教育、医疗、养老服务等方面供给还不充分，民生改善任务繁重；社会治理需要进一步加强，部分领域风险隐患不容忽视。政府自身建设还需加强，“放管服”改革有待进一步突破，行政效能有待进一步提高；作风建设长效机制还需进一步完善，干事创业的精神状态还需进一步提振。对此，我们将高度重视，并在今后工作中切实加以解决。

　　二、今后五年发展目标

　　党的十九大是在全面建成小康社会决胜阶段、中国特色社会主义进入新时代的关键时期召开的一次十分重要的大会。大会提出的一系列新判断、新要求、新部署，是我们做好当前以及今后一个时期工作的根本遵循和行动指南。我们要深入学习贯彻党的十九大精神，牢固树立“四个意识”，坚定“四个自信”，在政治立场、政治方向、政治原则、政治道路上同以习近平同志为核心的党中央保持高度一致，自觉维护以习近平同志为核心的党中央权威和集中统一领导，坚决把思想和行动统一到十九大精神上来，把智慧和力量凝聚到实现十九大确定的目标任务上来。

　　今后五年，是我国实现“两个一百年”奋斗目标的历史交汇期，也是我市加快建设“强富美高”新南京的关键期。我们要对标十九大部署要求，对标国内外先进城市，对标人民对美好生活的向往，以强烈的担当精神和使命意识，在发展定位和工作标准上确立更高的追求，奋力开拓新时代中国特色社会主义在南京实践的新境界。今后五年政府工作总的要求是：全面贯彻落实党的十九大精神，以习近平新时代中国特色社会主义思想为指导，按照总书记视察江苏重要讲话要求，坚持稳中求进工作总基调，坚持新发展理念，紧扣社会主要矛盾变化，按照高质量发展的要求，统筹推进“五位一体”总体布局和协调推进“四个全面”战略布局，围绕“两聚一高”“两高两强”和“一个高水平建成、六个显著”奋斗目标，立足“创新名城、美丽古都”城市定位，以推进供给侧结构性改革为主线，统筹做好稳增长、促改革、调结构、惠民生、防风险各项工作，打好防范化解重大风险、精准脱贫、污染防治三大攻坚战，高水平全面建成小康社会，高起点开启基本实现现代化建设新征程，加快建设“强富美高”新南京，努力在各项工作中走在全省前列、发挥表率作用。

　　今后五年全市经济社会发展的主要目标是：

　　——经济发展质量效益显著提升。经济发展质量变革、效率变革、动力变革取得明显成效，全要素生产率显著提高，经济创新力和竞争力显著增强。互联网、大数据、人工智能和实体经济深度融合，产业迈向价值链中高端，以先进制造业、现代服务业为主干的现代产业体系基本形成，“4+4+1”主导产业增加值占全市地区生产总值80%以上。供给侧结构性改革不断深化，传统产业转型升级取得明显成效，数字经济、人工智能等新技术、新产业、新业态、新模式快速发展，经济发展动能加快转换。军民技术相互支撑、有效转化，军民融合深度发展格局基本形成。

　　——创新名城建设取得重要突破。加快建设具有全球影响力的创新名城，建成国家创新型城市。综合性科学中心和科技产业创新中心建设取得重大进展，成为国家重大产业原创性技术成果和战略性新兴产业的重要策源地之一。创新创业生态系统位居全国一流，成为最鼓励、最适合、最具活力的创新创业城市之一。科技体制改革深入实施，“两落地一融合”取得显著成效，新型研发机构和高新技术企业数量大幅增加，创新型产业集群初步形成，名校名所名人名城融合发展进一步深化。建成国家知识产权强市。

　　——开放包容的现代都市形象全面展现。城市能级显著提升，基本建成首位度高的省会城市、影响力强的特大城市、国际化程度高的历史文化名城和幸福感强的宜居宜业城市。江北新区区域发展增长极的效应凸显，宁镇扬一体化和南京都市圈建设取得显著进展，扬子江城市群核心城市和龙头带动作用得到充分发挥，区域性经济、文化、金融、会展、商务功能更加完善，全国性综合交通枢纽和信息枢纽城市地位进一步提升，建成重要国际旅游目的地和国家旅游中心城市。开放型经济竞争力显著增强，城市功能设施体现国际水平，城市管理服务接轨国际标准，历史文化名城的国际影响力显著提升。

　　——城乡发展更趋融合。新型城镇化建设深入推进，江南江北、老城新城、主城郊区融合发展新格局基本构建。乡村振兴战略深入实施，城乡基础设施布局更加完善，公共服务资源配置更加均衡，努力把广大农村建成都市美丽乡村、农民幸福家园。现代农业产业体系、生产体系和经营体系不断健全，农村一二三产业进一步融合发展。宜居宜业的特色小镇充满活力，特色田园乡村建设品质持续提升，充分展现产业兴旺、生态宜居、乡风文明、治理有效、生活富裕的乡村发展新面貌。

　　——绿色南京魅力充分彰显。生态文明制度体系更加完善，生态产品供给持续增加，绿色低碳循环发展的经济体系不断健全。国土空间格局进一步优化，主体功能区布局和生态安全屏障格局基本形成。绿色南京建设持续推进，主要污染物排放总量大幅减少，空气质量优良天数比例达到80%以上，地表水优于Ⅲ类水质的比例大幅提升，土壤环境得到有效管控，城市生活垃圾分类收集全覆盖，打赢污染防治攻坚战，生态环境质量显著改善，南京的天更蓝、水更清、地更绿、环境更优美。

　　——民生福祉持续增进。人民群众有更满意的收入、更好的教育、更稳定的工作、更可靠的社会保障、更高水平的医疗卫生服务、更舒适的居住条件、更优美的环境、更丰富的精神文化，获得感、幸福感、安全感更加充实、更有保障、更可持续。居民人均可支配收入与全市经济同步增长，城乡居民收入差距进一步缩小。符合标准的低收入人口全面脱贫、经济薄弱村全部摘帽。15分钟公共服务圈全面建成。基本实现教育现代化，义务教育优质资源覆盖率达到100%。健康南京建设取得重大进展。新型住房保障体系、多层次养老服务体系基本形成。

　　——社会文明程度和治理能力进一步提高。中国梦和社会主义核心价值观深入人心、有效践行，市民思想道德素质、科学文化素质、健康素质明显提高，社会诚信体系基本建立。现代公共文化服务体系不断完善，公共文化服务标准化均等化程度达到85%以上。文化软实力显著增强，现代文化产业体系和市场体系进一步健全，文物保护利用和文化遗产保护传承进一步加强。公共安全水平明显提高，市民百姓安全感保持在92%以上。共建共治共享的社会治理模式加快形成，城市治理体系和治理能力现代化取得重大进展。全社会法治意识不断增强，依法行政和法治政府建设处于全国全省前列。

　　实现上述目标，我们必须坚定不移以习近平新时代中国特色社会主义思想为指导，更加自觉地用习近平新时代中国特色社会主义思想武装头脑、指导实践、推动工作；坚定不移贯彻以人民为中心的发展思想，更大力度办好民生实事，不断满足人民日益增长的美好生活需要，切实增强市民百姓的获得感、幸福感和安全感；坚定不移推动高质量发展，努力实现经济发展、改革开放、城乡建设、文化建设、生态环境和人民生活高质量，奋力开创南京改革发展新局面；坚定不移对标找差、创新实干，在更高坐标系中提升发展标杆、确立更高追求，充分发挥好省会城市辐射带动力，提高城市首位度；坚定不移加强政府自身建设，不断提升治理能力现代化水平，增强政府公信力和执行力，加快建设人民满意的服务型政府，努力把习近平总书记为我们描绘的“强富美高”蓝图变成美好现实。

　　三、2018年经济社会发展主要任务

　　2018年是贯彻党的十九大精神的开局之年，是改革开放40周年，是决胜全面建成小康社会、实施“十三五”规划承上启下的关键一年，也是新一届政府履行职责的第一年，做好政府工作至关重要。必须贯彻落实中央经济工作会议和市委十四届五次全会精神，在高水平全面建成小康社会、高质量推进“强富美高”新南京建设方面取得扎实进展、迈出更大步伐。

　　今年全市经济社会发展的主要目标分为两类：一是预期性指标。地区生产总值增长8%；固定资产投资增长10%，其中高新技术产业投资增长12%，工业投资增长10%，工业技改投资占工业投资比重提高2个百分点；社会消费品零售总额增长10%；外贸进出口总额增长3%，服务贸易进出口总额增长10%以上，实际利用外资38亿美元；一般公共预算收入增长8%；建设用地地均GDP达41万元/亩；新增规模以上工业企业200家，新增高新技术企业800家；全社会研发投入占GDP比重达到3.1%，规模以上工业企业研发投入占主营业务收入的比重达1.5%；高新技术产业产值占规模以上工业总产值比重提高0.5个百分点，战略性新兴产业增加值占GDP比重达18%；全体居民人均可支配收入增长8%以上，农村居民人均可支配收入增速高于城镇居民人均可支配收入增速；居民消费价格指数控制在省定目标以内，城镇新增就业24万人，城镇调查失业率和登记失业率分别控制在省定标准和3.5%以内。二是约束性指标。全市万元GDP能耗下降4%，煤炭消费总量减少250万吨以上；万元GDP用水低于34立方米；主要污染物排放持续下降、达到省定标准，万元GDP二氧化碳排放下降4%；空气质量优良天数比例达75%；全市域基本消除黑臭水体，地表水省考以上断面好于Ⅲ类水质比例达63.6%。

　　今年，我们要围绕推动高质量发展，做好九项重点工作：

　　（一）坚持聚力创新，加速转换发展动能

　　把创新作为引领发展的第一动力，大力实施创新驱动发展“121”战略，加快推进“两落地一融合”工程，不断完善城市创新创业生态系统，推动科技创新成为高质量发展的战略支撑。

　　夯实创新驱动发展基础。启动综合性科学中心建设，加强重大科技创新平台建设，争取一批国家重大专项落地。全面深化与高校院所的战略合作，推进科技成果产业化、项目化，推动南京智能制造研究院等一批新型研发机构建设，确保全市新型研发机构达到100家。加快科技产业创新中心建设，推动15个高新园区管理机构和运作平台实质性运作，突出创新发展、特色发展、集聚发展，加快培育具有国际竞争力的产业创新集群。制定高新园区考核办法，配套相关政策，为科技成果项目和新型研发机构落地提供更加优质的创新熟地。加快推进南京麒麟科技城、省产业技术研究院等一批重大项目建设，为江苏建设产业科技创新中心贡献南京力量。

　　强化企业创新主体地位。大力实施科技企业培育工程，建立科技型中小微企业、高新技术企业、瞪羚企业、独角兽企业培育库，加大服务支持和资源倾斜力度，把一批科技含量高、创新能力强、成长性好的科技型中小企业培育成为高新技术企业。大力实施企业研发能力提升工程，支持大中型工业企业和规模以上高新技术企业建设研发平台，简化研发费用加计扣除等科技政策落实手续；鼓励离岸创新创业企业并购或设立海外研发机构。做强企业服务中心，为创新型企业提供一站式综合服务。全年建设市级以上企业研发机构超过100家，新增省级企业研发机构不少于30家。

　　构建优良创新创业生态系统。深化科技体制综合改革，充分激发科技人员的积极性。加大科技财政投入，设立市级科技创新基金，支持金融机构在宁集聚，发展天使基金、种子基金，推动科技与金融深度融合。推进实施“创业南京”英才计划、海外人才“345”引进计划、中青年拔尖人才选拔培养计划和青年大学生“宁聚计划”等重点工程，全力支持企业引进培育高端人才。进一步加大人才安居保障力度，优化人才落户政策，推动人才服务国际化。进一步推进大众创业、万众创新，国家级科技企业孵化器和众创空间达到90家以上。推进智能生态岛建设。加强知识产权创造、保护与运用，大力推进开放创新，支持创新产品推广应用，建立科技创新容错机制，打造充满活力、更具竞争力的创新创业生态系统。

　　（二）加快转型升级，着力建设现代产业体系

　　深化供给侧结构性改革，坚持先进制造业和现代服务业“双轮驱动”，推动“4+4+1”主导产业优化升级，加快建设主导产业清晰、比较优势明显、集聚效应突出的产业体系，切实增强经济创新力和竞争力。

　　推动先进制造业集群发展。按照习近平总书记视察江苏时提出的“必须始终高度重视发展壮大实体经济，抓实体经济一定要抓好制造业”要求，以“中国制造2025”试点示范城市建设为抓手，大力推动制造业高端化、智能化、绿色化、集聚化发展，加快构筑南京未来发展新优势。坚持有限目标、重点突破，进一步整合现有产业资源，优化产业空间布局，聚力打造新型电子信息、绿色智能汽车、高端智能装备、生物医药与节能环保新材料四大先进制造业主导产业，努力形成规模优势领先、核心技术领先、产业配套完善的产业集群，主营业务收入增长12%以上。把握科技和产业发展趋势，前瞻布局人工智能、未来网络、增材制造与前沿新材料、生命健康等未来产业，努力赢得产业升级的先机和主动。创建全国质量提升示范区。加快发展军民结合产业，力争“民参军”企业达到150家。全力推动先进制造业重大项目建设，开工建设紫光集团存储器制造基地、中兴通讯智能制造全球总部等一批百亿级项目，推动台积电一期、FMC智能电动汽车等一批龙头项目顺利投产，为制造业发展提供坚实支撑。

　　推动现代服务业高端发展。大力推进软件和信息服务、金融和科技服务、文旅健康、现代物流与高端商务商贸等现代服务业主导产业集中集聚发展，加快发展新金融、新零售等新业态新模式，不断提高现代服务业发展的层次水平。围绕国际软件名城建设目标，聚焦新一代软件和信息服务业发展重点领域，进一步加大政策支持、载体建设、产业集聚力度，持续提升产业发展竞争力。加快推进金融城二期等一批金融龙头项目，全力建设以“一江两岸”为核心的泛长三角区域金融中心。强化多层次资本市场发展和利用，力争新增上市企业数超过上年。以满足科技创新需求和提升产业创新能力为导向，加快发展科技服务业，打造有全国影响力的科技服务业中心城市。整合全市文化旅游资源，加快实施“文化+”战略，推动旅游集团做强主业，加快国家全域旅游示范区建设。与群众健康需求深度对接，进一步提升健康服务和养老产业供给水平。加快实施阿里巴巴江苏总部等一批重大项目，着力推动服务业向价值链高端攀升。

　　深入实施“三去一降一补”。加快传统产业转型升级，推动南化、南钢、梅钢等重点企业布局调整和转型发展取得实质性进展，完成年度钢铁去产能任务，化解船舶产能20万载重吨。坚持“房子是用来住的、不是用来炒的”定位，加快建立多主体供给、多渠道保障、租购并举的住房制度，保持房地产市场平稳健康发展。发展住房租赁市场特别是长期租赁，保护租赁利益相关方合法权益，支持专业化、机构化住房租赁企业发展，优化土地供给结构，加大租赁住房建设力度，租赁房供地占住房供地比例达到30%以上。有效防范化解金融风险，促进形成金融和实体经济、金融和房地产、金融体系内部良性循环，做好重点领域风险防范和处置，严密排查、严厉打击非法集资等违法违规金融活动，严格互联网金融市场准入，积极稳妥做好金融风险个案稳控化解工作，设立金融监督管理局。加强政府债务管理，有效防控化解政府债务风险。落实好降低企业成本各项政策，加大对乱收费的查处和整治力度，为企业降低成本200亿元左右。加快推动补短板重点项目，着力提高经济社会发展的协调性和均衡性。

　　（三）蹄疾步稳全面深化改革，不断提升开放水平

　　坚定不移深化改革、扩大开放，为全市经济社会发展注入更多的动力和活力。

　　打好全面深化改革攻坚战。深入推进行政审批制度改革，加快推广“不见面审批”和“3550”改革，全面推开“双随机、一公开”监管，深化“互联网+政务服务”建设，“我的南京”APP集成更多服务功能，让企业办事更方便、群众办事更舒心。深化商事制度改革，推行企业登记“多证合一、一照一码”，在国家级开发区和江北新区开展“证照分离”改革试点，全面实施市场负面清单制度。进一步激发各类市场主体活力，加快国企混合所有制和多元化改革，推动国有资本做强做优做大；支持民营企业发展，落实保护产权政策，推动民营企业转型升级，鼓励民营资本进入基础设施、公共服务等领域，力争民营经济增加值增长8%。统筹推进科技人才、贸易流通、生态、民生等各领域改革，进一步增强改革的系统性、整体性、协同性。突出抓好国家和省重大改革试点，支持江宁区开展集成改革试点工作。

　　构建对外开放新格局。把引进外资作为调结构转方式、促进高质量发展的重大举措，坚持引资引技引智并举，着力招引先进生产要素和大项目好项目，带动南京企业嵌入全球产业链、价值链和创新链。加快培育外贸新业态新模式，在稳住外贸基本面的同时，着力提升出口产品质量和附加值。对标自由贸易区、自由贸易港，进一步壮大服务贸易、服务外包和跨境电子商务规模，着力培育以技术、品牌、质量、服务为核心的外贸竞争新优势，保持外贸进出口良好势头。积极参与“一带一路”国际合作，加快推动装备、技术、标准、服务“走出去”。推动开发园区转型升级、创新发展，着力打造特色创新集群。推动三大枢纽经济区提速发展，新开通南京至赫尔辛基等一批国际客货运航线，着力提升机场、港口和铁路吞吐能力。加快电子口岸平台建设，切实提升口岸便利化、国际化水平。加强宁台合作交流，推动宁台产业融合发展。继续打造好金洽会、软博会、全球服务贸易大会等一批重大开放平台，在更大范围内集聚资源要素。办好2018年世界羽毛球锦标赛、世界女排联赛总决赛等一批重大体育赛事，精心筹备2020年世界室内田径锦标赛，提升世界体育名城影响力。

　　积极推动区域协调发展。抢抓长江经济带发展机遇，发挥好南京在扬子江城市群建设中的龙头作用，夯实区域融合发展基础。聚焦基础设施建设、产业发展、公共服务、毗邻地区发展等重点，加快跨区域轨道交通、创新合作等一批项目建设，推动宁镇扬一体化全面提速。积极参与长三角一体化发展，加快推动宁杭生态经济带建设。扎实推进援藏、援疆、扶贫协作陕西商洛和青海西宁，做好对口支援重庆万州和宁鞍合作、宁淮挂钩工作。

　　（四）狠抓规划建设管理，提升城市承载能力

　　积极顺应城市发展规律，进一步完善城市功能、提升城市品质，加快建设幸福感强的宜居宜业城市。

　　坚持规划引领城市发展。高水平推进新一轮城市总体规划修编，科学谋划确定城市战略定位、空间格局和要素配置，深入实施主体功能区规划，全面提升国土空间开发保护质量和效率，坚持城乡统筹、落实“多规合一”，形成一本规划、一张蓝图，强化规划的战略引领和刚性控制作用。落实老城建筑高度规划管理规定，严格控制历史城区、历史地段、明城墙沿线、景观视线走廊的建筑高度。认真做好城市设计试点工作，加强对城市街道设计、建筑设计、城市色彩的控制引导，进一步塑造生态人文空间，强化城市“山水城林”特色。

　　加快构建综合交通体系。推进交通基础设施互联互通，提升综合运输服务质量效率。开工建设南京北站综合客运枢纽，加快推进龙潭疏港公路、西坝港区铁路专运线等集疏运体系建设，进一步完善南京交通枢纽功能。大力推进对外交通建设，加快推进溧阳至高淳高速、宁合高速改扩建、宁马高速改扩建等一批道路建设。新开工及续建干线公路483公里。持续保持轨道交通建设势头，同步推进5号线、6号线、7号线等9条地铁线路建设，确保宁溧线建成通车。实施城市交通堵点治理攻坚，推进红山路—和燕路快速化改造、光华路东延、岱山东路北延、机场二通道等一批道路工程，开工建设绕城公路徐庄互通，推动城市快速路与高速公路共同形成“井字三环、轴向放射、组团快联”路网格局，让城市更加畅通、市民出行更加便捷。

　　统筹优化城乡空间布局。始终坚持“老城做减法、新城做加法”的城市发展思路，切实加强古都风貌保护，积极实施“城市双修”，加快推动非古都核心功能向外围新区布局，推动实现江南江北、老城新城、主城郊区协同发展。加快推进河西新城、南部新城、江心洲生态科技岛、麒麟高新区建设与发展，不断提升仙林副城、东山副城、溧水副城、高淳副城城市品质，优化铁心桥—西善桥、鼓楼滨江、玄武铁北等片区整体环境，尽快成为各具特色、功能完善、职住平衡、产城融合的新高地。坚持节约集约用地，实施产业用地绩效评价，盘活城镇存量用地2.1万亩。

　　加强城市精细化管理。深入推进城市精细化建设管理十项行动，实施主次干道环境综合整治和背街小巷整治；切实加强“四沿”环境整治提升，实施一批绿化亮化提升工程，持续提升城市“颜值”。加强城市立体式开发，统筹推动地下综合管廊和海绵城市建设，城市地下空间开发利用总量达3700万平方米，建成区5%的面积达到海绵城市建设要求。加强智慧南京建设，在城市治理、公共服务等方面提供更加便捷高效的智慧应用。深化城市治理，加大共享单车管理、城市环卫保洁、停车管理、打非拆违等工作力度，实施农贸市场提档升级。提升城市供水、供气、供电等保供能力。加快垃圾分类处理基础设施建设，党政机关、事业单位率先推行生活垃圾强制分类。坚持不懈推进“厕所革命”，加快城市公共厕所提档升级，分批推进农村公共厕所改造。

　　（五）实施乡村振兴战略，推动城乡融合发展

　　按照产业兴旺、生态宜居、乡风文明、治理有效、生活富裕的总要求，大力推动乡村振兴，加快推进农业农村现代化。

　　推进农业供给侧结构性改革。坚持质量兴农、绿色兴农，进一步优化农业产品结构、产业结构、布局结构，加快构建现代农业产业体系、生产体系、经营体系，提高农业发展质量效益和竞争力。完善现代农业经营体系，加快培育新型经营主体和新型职业农民，积极发展家庭农场和农民专业合作社，做大做强农业龙头企业，健全农业社会化服务体系。提升农业科技和装备应用水平，大力发展生物农业、智慧农业等都市型现代农业，加快发展特色产业、休闲农业、乡村旅游、农村电商等新业态新模式，延伸农业产业链、价值链，推动一二三产业深度融合。推进农业绿色发展，实施畜禽养殖污染及农业面源污染专项治理。建设各类农业载体平台，高标准打造南京国家现代农业产业科创园，支持白马高新区等40个重点农业园区加快发展。全年建设高标准农田7万亩、设施农业2万亩，农业机械化水平达86.5%，农业信息化水平达61%。巩固提升粮食产能，全面保障粮食安全。

　　推进特色田园乡村建设。根据不同地区和乡村的个性特色，注重保护乡村传统肌理、空间形态和传统建筑，做好重要空间、建筑和景观设计，保留乡村风貌，保护生态要素，留住田园乡愁，全年建设美丽乡村示范片区300平方公里、示范村80个、田园综合体5个。深化农村环境综合整治，扎实做好垃圾处理、污水处理、绿化美化等工作。加快推进农村水利和交通基础设施建设，完成3.5万亩高效节水灌溉工程建设，实施农村公路提档升级。加强农村基层基础工作，健全自治、法治、德治相结合的乡村治理体系，大力推进农村精神文明建设，提升农民精神风貌，让乡村成为市民向往之地、农民幸福家园。

　　健全城乡融合发展的体制机制。完善规划体制，通盘考虑城乡发展规划编制，通过改革的办法、创新的举措，加快建立城乡统一的人才、土地、科技、资本等要素市场，引导和推动更多的要素向农业农村流动，促进城乡共同繁荣。进一步完善农村公共服务，加快农村教育卫生等社会事业发展，促进城乡基础设施互联互通、共建共享。探索“三权分置”多种实现方式，实施农村土地综合整治，加大集体建设用地建设租赁用房力度，进一步激发农村发展活力。扎实推动农村集体产权制度改革，扩大农村集体产权股份合作制改革试点范围，切实盘活农村集体资产、增加农民财产性收入、发展壮大集体经济。统筹推进农业支持保护制度等领域改革，加强农村集体资金、资产、资源管理专项治理。大力发展农村普惠金融。积极开展农业水价综合改革，促进农业用水和农田水利工程良性运行。继续深化经济发达镇行政管理体制改革。

　　（六）强力推进江北新区建设，打造新的增长极

　　牢牢把握“三区一平台”战略定位，进一步聚焦重点、突破难点、抓住关键点，尽快把江北新区建设成为全省未来创新策源地、引领区和重要增长极。

　　强化新区产业支撑。落实好江北新区创新创业十条政策，支持新区在人才引进、研发机构落地、创新创业等方面创新政策，着力打造全省人才高地、创新高地和产业高地。加速集聚海内外高端创新资源，建设好中德智能制造示范园区、中法产业合作示范区等一批创新平台，着力建设具有全球影响力产业科技创新中心的重要基地。全力打造集成电路、生物医药、新能源汽车等三大千亿级产业集群，加快推进产业技术研创园等重点产业平台建设，积极引进一批龙头项目，不断完善产业链条。着力推动现代金融要素集聚，加快建设基金城、金融科技园和资产管理中心，加速形成新金融业态集聚效应。大力发展现代物流、科技服务等现代服务业，支持新区申报设立南京江北海港枢纽经济区保税物流中心。积极推进服务贸易创新发展试点，加快发展大数据、信息服务等新兴服务贸易，形成一批可复制可推广的经验。扩大有效投入，强化项目引领，加快推进一批龙头项目建设，力争新区主要经济指标增速高于全市平均增速5个百分点以上。

　　加快新区城市建设。高标准推动新区核心区建设，以新区研创园、中央商务区、国际健康城为开发建设重点，加快推进绿地金融中心、服务贸易创新大厦等一批地标性项目建设，有序推进新区规划展览馆、南京美术馆新馆、江北图书馆新馆等一批功能性项目，尽快展示新区新形象，打造城市新名片。积极推动事关新区长远发展的一批重大基础设施建设，开工建设宁天线南延工程，启动地铁4号线二期、11号线一期两条江北新线建设。加快过江通道建设，完成长江大桥公路桥维修改造，推进长江五桥、浦仪公路西段、和燕路过江通道南段建设，新开工仙新路过江通道，做好建宁西路过江通道准备工作。落实规划新理念、新技术，加快新区CBD地下空间、综合管廊、海绵城市、青龙绿带、骨干路网建设，提升新区城市治理水平，为长远发展夯实基础。

　　推动新区改革创新。优化新区管理体制机制，理顺新区直管区、共建区和协调区关系，发挥好高新片区在苏南国家自主创新示范区建设中的主力军作用，积极推动江北新区新材料科学园转型发展，加快浦口区、六合区发展步伐。进一步构建高效行政管理体制，加快探索高位协调、扁平化管理、项目化市场化运作、激励性考核评价运行模式。承接好46项省级管理事项权限和市级管理权限，加快实施相对集中行政许可权改革，打造行政审批事项最少、办结时限最短、收费最少的国家级新区。

　　（七）践行绿色发展理念，加快建设美丽南京

　　牢固树立“绿水青山就是金山银山”理念，深入落实生态环境保护工作责任，加快建设人与自然和谐共生的生态城市。

　　全力治理突出环境问题。全面实施《生态环境保护工作责任规定》，扎实开展“263”专项行动，重点推进减煤、减化、减铸造，全年关停化工企业40家、整治铸造企业42家。深化大气污染防治，加强挥发性有机物治理，打好餐饮油烟污染治理攻坚战，加强施工机械排气管控，加大工业废气、工地扬尘、秸秆焚烧污染治理力度，让群众真切感受到空气质量一年更比一年好。进一步加大治水力度，聚焦污水收集管网及处理设施建设、水体整治提升、片区雨污分流三大重点，实施水环境提升工程，启动建成区消除劣Ⅴ类水体行动，完成87平方公里雨污分流改建工程，建成400个规划布点村污水处理设施；严格落实“河长制”，健全完善河道、排水排污管网分级管理长效机制，打造水清岸绿景美的城市水环境。加强水源地保护，确保区级以上集中式饮用水水源地水质达标率保持100%。全面实施“土十条”，建立污染地块名录及其开发利用负面清单，加强土壤污染防治，改善土壤环境质量，保障土壤环境安全。

　　加大生态建设力度。严格落实“共抓大保护，不搞大开发”要求，加强流域治理，加大长江岸线生态保护力度，基本消除外秦淮河、金川河等主要入江支流劣Ⅴ类水质断面。严格落实生态红线刚性保护，实施生态红线区域清理整治，加大宕口整治力度。实施新一轮城乡绿化行动计划，全市林木覆盖率达到30.5%。实施城区园林景观提升工程，完成一批行道树彩色化景观提升，推动中山陵、玄武湖景区环境整治提升，推进红山森林动物园、石头城遗址公园、仙林湖公园等一批公园改造以及明外郭—秦淮新河景观走廊建设，不断彰显城市绿色之美、生态之美。

　　健全生态文明体制机制。实行以“产业准入、区域准入”为核心的最严格环境准入制度，建立环境质量和污染总量双控管理机制，全面实行排污权有偿取得和交易、生态补偿等环境价格机制，实施与污染物排放总量挂钩的财政政策，着力形成节约资源和保护环境的产业结构、生产方式。有序推进环保监测监察执法机构垂直改革，提升环保执法监管水平，坚决制止和惩处破坏生态环境行为。扎实推进领导干部自然资源资产离任审计，健全绿色发展考评体系，落实损害生态环境终身追责制度。

　　（八）坚定文化自信，建设文化强市

　　进一步坚定文化自信、打造文化标识、讲好南京故事、建好精神家园，把文化强市建设推向新的高度。

　　提升城市文明程度。深入开展中国特色社会主义和中国梦教育，坚定市民“四个自信”，使全市人民在理想信念、价值理念和道德观念上紧紧团结在一起。大力弘扬社会主义核心价值观，切实用社会主义核心价值观凝心聚力，用中华传统美德成风化俗，构筑道德风尚建设高地。制定实施《南京红色文化资源保护利用规划》，挖掘南京丰富的党史资源，大力传承弘扬雨花英烈精神，把红色基因一代代更好地传承下去。办好南京大屠杀死难者国家公祭仪式、纪念改革开放40周年等一批重大活动。实施公民道德建设工程，进一步巩固文明城市创建成果，深化群众性精神文明创建活动，推动形成向上向善的良好社会风尚。推进新型智库建设，深化哲学社会科学理论研究。加强互联网建设和管理，营造清朗网络空间。

　　加强历史文化名城保护。传承利用好南京丰富的历史文化遗产，让城市散发出更多文化的温度。加强明城墙修缮保护利用，加快城墙博物馆建设，推进南京城墙申遗工作，打造世界级城市文化名片。加强近现代建筑保护利用和功能转换，对秦淮民居群等一批文物实施修缮。推动非物质文化遗产活化利用，办好2018年“文化和自然遗产日”主题活动。推进科举博物馆二期建设，整合全市文博场馆资源，打造“博物馆之城”。加强国际传播能力建设，向世界讲好南京故事。推进和平城市建设，继续办好南京历史文化名城博览会和世界知名城市“南京周”活动，加大城市对外宣传推介力度，提升南京历史文化名城国际影响力。

　　推动文化事业和文化产业发展。加快构建现代公共文化服务体系，深入实施文化惠民“百千万行动计划”，办好南京森林音乐会、南京文化艺术节等重点文化品牌活动。实施文艺精品创作工程，制定文艺创作规划，评选“南京市文学艺术奖”，为群众提供丰富的精神食粮。大力推进全民阅读，积极推进“书香南京”建设，推动公共图书馆、文化馆、美术馆等免费开放，加快公共文化服务数字化建设步伐。建设创意文化产业功能区，打造特色化、品牌化园区基地，提高文化产业规模化、集约化、专业化水平。抓好南京新媒体大厦等重点文化工程建设，推进文化消费试点，发展出版传媒、影视动漫、广告会展、文化创意等优势特色产业，办好中国南京文化创意产业交易会，推动文化与科技、金融、旅游、互联网深度融合发展。推进实施金梧桐文化企业30强培育计划，发展骨干龙头文化企业，持续提升城市文化软实力。

　　（九）切实保障和改善民生，加强和创新社会治理

　　着力解决结构性民生问题、实施普惠性民生工程、办好扶助性民生实事、满足多样化民生需求，努力在发展中进一步提高保障和改善民生水平。

　　加快富民增收步伐。始终坚持产业富民、创业富民、政策富民，积极促进农民等重点群体增收，加快建立与经济发展相适应的收入增长机制，推动居民收入增长与经济发展同步。大力发展高新技术产业、高增加值产业和品牌经济，加快发展特色富民产业，通过产业层次提升带动居民收入增长。实施更加积极的创业就业政策，注重解决结构性就业矛盾，打造对青年大学生就业创业最具吸引力城市，确保新增大学生就业20万人以上，培育自主创业者1.5万人。充分调动技能人才、新型职业农民、科技人员等重点群体的积极性、创造性和带动性，实行差别化的收入分配政策，有效扩大中等收入群体比重。深入实施脱贫致富奔小康行动计划，做好建档立卡低收入农户家庭增收工作，促进经济薄弱村稳定性收入达到100万元。

　　推动基本公共服务标准化均等化。把公共服务作为最大普惠，坚持问题导向、增加服务供给、创新提供方式，落实好《“十三五”时期基层基本公共服务功能配置标准（试行）》，提升11个领域107项公共服务供给质量。落实立德树人根本任务，提升素质教育发展水平；加强义务教育学校标准化建设，力争义务教育优质资源覆盖率达95%；基本消除“大班额”现象，进一步缓解“择校热”，切实减轻中小学生过重课外负担；增加公办幼儿园供给，加强幼儿教师队伍建设；优化普通高中布局，推动5所市属高中在新城新区扩建，与本部一体化办学；加强学校安全风险防控管理，在全市所有中小学和幼儿园设置常态化固定护学岗，切实保障中小学生和幼儿安全健康。进一步优化医疗资源布局，实现鼓楼医院江北医疗中心试运行；提升基本公共卫生均等化水平，深化医疗联合体、家庭医生、智慧医疗建设，为群众提供更加便捷的医疗服务。鼓励社会资金进入医疗、养老等领域，大力推进居家养老、医养融合。加强残疾人之家建设。

　　织密扎牢民生保障网。按照兜底线、织密网、建机制的要求，深化社会保障领域改革，全面实施全民参保计划，进一步完善社会保险市级统筹，扩大企业年金制度覆盖面。建立全市统一的城乡居民医保制度，提高居民医保财政补助标准，提高大病保险待遇水平。做好退休人员、特殊群体养老保险待遇调整工作，进一步提高城乡低保标准。加强住房保障体系建设，落实好城市低收入困难家庭、新就业大学生住房租赁补贴。进一步做好老旧小区整治、积淹水片区整治改造等工作。

　　打造共建共治共享的社会治理格局。全面推进创新网格化社会治理机制试点工作，深入开展全国社区治理和服务创新试验区建设。加强社会心理服务体系建设，培育自尊自信、理性平和、积极向上的社会心态。深入扎实推进“大走访”活动，积极化解社会矛盾，支持工青妇等群团组织参与社会治理。强化信用大数据应用，切实推进“诚信南京”建设。深化国防动员和双拥共建，巩固军政军民团结。加强食品药品安全监管，创建国家食品安全示范城市。全面落实安全生产责任制，提高防灾救灾能力，提升高层建筑消防能力，坚决遏制重特大安全事故。深化现代化立体化信息化社会治安防控体系建设，加大对各类犯罪活动的惩治力度，坚决打击群众反映强烈的网上虚假信息诈骗、倒卖个人信息等违法行为，保障群众生命财产安全。

　　保障和改善民生要抓住人民群众最关心最直接最现实的利益问题。今年，我们在广泛征集民意的基础上，围绕群众最关心的公共服务、城市品质、社会保障、居民增收、交通出行、文体惠民、社会安全等七个方面，再办好35件民生实事，不断增强广大群众的获得感、幸福感、安全感。

　　四、建设人民满意的服务型政府

　　新时代标注新方位，新使命要有新作为。站在城市发展的新起点，踏上未来五年的新征程，新一届政府将勤勉履职、奋发作为，加快转变政府职能，持续深化简政放权，不断创新监管方式，进一步增强政府公信力和执行力，切实做到对人民负责、受人民监督、让人民满意。

　　（一）坚持为民勤政，建设开拓创新、务实高效的政府。坚持把人民对美好生活的向往，作为新一届政府奋斗目标，做到与人民同呼吸、共命运、心连心。坚持目标导向、问题导向、攻坚导向、变革导向，坚持系统化思维、注重规律性把握，着力增强改革创新、科学发展、依法行政、群众工作、狠抓落实、驾驭风险的本领，不断开创发展新局面。坚持创新发展理念，全面开展对标找差、创新实干工作，加快形成高质量发展的指标体系、政策体系、标准体系、统计体系、绩效评价、政绩考核。完善重点工作推进机制，坚持从严管理与正向激励相结合、容错与纠错相结合，充分调动各级干部干事创业的积极性、主动性和创造性。坚持实干为民，改进和创新工作方法，确保工作项目化、项目目标化、目标节点化、节点责任化，在实干中补齐差距、在创新中塑造优势。大力弘扬“马上就办”作风，以“今天再晚也是早、明天再早也是晚”的精神，发挥真抓的实劲、敢抓的狠劲、善抓的巧劲、常抓的韧劲，抓铁有痕、踏石留印，进一步提升执行力和落实力。落实好新南京建设工作联系点制度，深入调查研究，强化高位协调，着力解决好发展中的不平衡不充分问题。

　　（二）坚持依法行政，建设厉行法治、规范用权的政府。实施法治政府建设五年规划，落实政府权力和责任清单，严格依法依规履职。健全依法决策机制，严格落实法定决策程序，制定公众参与重大行政决策程序操作规则，实现市区两级政府及部门法律顾问制度全覆盖，切实提升决策科学化、民主化水平。坚持严格规范公正文明执法，加快探索跨部门、跨区域综合执法，推动行政执法和刑事司法有效衔接。自觉接受市人大及其常委会法律监督和工作监督、政协民主监督，认真执行市人大及其常委会决议决定，坚持重大事项向市人大及其常委会报告、向市政协通报制度，健全与人大代表、政协委员的沟通联络机制，高质量办好建议提案，广泛听取各民主党派、工商联、无党派人士和人民团体意见。全面推进政务公开，建成政府数据统一开放平台，有序推进政府数据开放共享。科学组织“十三五”规划中期评估。精心开展第四次全国经济普查。

　　（三）坚持从严治政，建设政治坚定、清正廉洁的政府。坚持旗帜鲜明讲政治，将全面从严治党要求贯穿到政府工作全过程、各方面，坚决落实党中央国务院、省委省政府和市委的各项决策部署，确保政令畅通、令行禁止。深入推进“两学一做”学习教育常态化制度化，扎实开展“不忘初心、牢记使命”主题教育。严格执行准则条例，自觉践行“三严三实”，坚决反对“四风”，深入查摆形式主义、官僚主义新表现，着力解决表态多调门高、行动少落实差等问题，以高标准严要求巩固拓展落实中央八项规定精神成果。坚持政府过“紧日子”，进一步加强政府支出管理，扎实有序推进审计监督全覆盖，支持人大开展预算联网监督，严控“三公”经费和一般性支出规模。深入落实党风廉政建设责任制，切实构建“亲”“清”新型政商关系，严查群众身边的腐败问题，大力营造风清气正的干事创业环境，保持各级政府的廉洁本色和良好形象。

　　各位代表：新征程鼓舞人心，新任务催人奋进。让我们更加紧密地团结在以习近平同志为核心的党中央周围，高举中国特色社会主义伟大旗帜，以习近平新时代中国特色社会主义思想为指导，在省委省政府和市委的坚强领导下，不忘初心、牢记使命，锐意进取、埋头苦干，加快推进“两聚一高”“一个高水平建成、六个显著”新实践，为建设“强富美高”新南京、高水平全面建成小康社会而努力奋斗！
